# Estimated Cancer Risks Associated with Nitrosamine Contamination in Commonly Used Medications

**DOI:** 10.3390/ijerph18189465

**Published:** 2021-09-08

**Authors:** Kate Li, Karin Ricker, Feng C. Tsai, ChingYi J. Hsieh, Gwendolyn Osborne, Meng Sun, M. Elizabeth Marder, Sarah Elmore, Rose Schmitz, Martha S. Sandy

**Affiliations:** 1 Office of Environmental Health Hazard Assessment (OEHHA), California Environmental Protection Agency, Oakland, CA 94612, USA; Kate.li@oehha.ca.gov (K.L.); Karin.ricker@oehha.ca.gov (K.R.); Feng.Tsai@oehha.ca.gov (F.C.T.); Gwendolyn.Osborne@oehha.ca.gov (G.O.); Sarah.Elmore@oehha.ca.gov (S.E.); 2 Office of Environmental Health Hazard Assessment (OEHHA), California Environmental Protection Agency, Sacramento, CA 95812, USA; Jennifer.Hsieh@oehha.ca.gov (C.J.H.); Meng.Sun@oehha.ca.gov (M.S.); Elizabeth.Marder@oehha.ca.gov (M.E.M.); Rose.Schmitz@oehha.ca.gov (R.S.)

**Keywords:** cancer, nitrosamines, risk assessment

## Abstract

Many nitrosamines are potent carcinogens, with more than 30 listed under California’s Proposition 65. Recently, nitrosamine contamination of commonly used drugs for treatment of hypertension, heartburn, and type 2 diabetes has prompted numerous Food and Drug Administration (FDA) recalls in the US. These contaminants include the carcinogens NDMA (N-nitrosodimethylamine) and NDEA (N-nitrosodiethylamine) and the animal tumorigen NMBA (N-nitroso-N-methyl-4-aminobutyric acid). NMBA and NDEA are metabolically and/or structurally related to NDMA, an N-nitrosomethyl-*n*-alkylamine (NMA), and 12 other carcinogenic NMAs. These nitrosamines exhibit common genotoxic and tumorigenic activities, with shared target tumor sites amongst chemicals and within a given laboratory animal species. We use the drug valsartan as a case study to estimate the additional cancer risks associated with NDMA and NDEA contamination, based on nitrosamine levels reported by the US FDA, cancer potencies developed by California’s Proposition 65 program and the US Environmental Protection Agency (EPA), and specific exposure scenarios. These estimates suggest that nitrosamine contamination in drugs that are used long-term can increase cancer risks and pose a serious concern to public health.

## 1. Introduction

In July 2018, the US Food and Drug Administration (US FDA) announced the recall of several drug products containing valsartan, a widely used angiotensin II receptor blocker (ARB), after the detection of the nitrosamine N-nitrosodimethylamine (NDMA) [1]. Shortly thereafter, NDMA and other nitrosamines were also detected in additional ARBs (losartan, irbesartan), certain histamine-2 blockers (ranitidine, nizatidine) used to treat heartburn and gastroesophageal reflux disease (GERD), and in an antihyperglycemic medication (metformin) used to treat type 2 diabetes. By July 2021, US FDA had announced over 60 recalls of contaminated prescription and over-the-counter (OTC) drug products from different manufacturers [2]. More recently, US FDA issued the first voluntary recall alert for the antismoking drug varenicline following detection of another nitrosamine, N-nitroso-varenicline [3]. Similar recalls of these types of drugs due to nitrosamine contamination have occurred in Japan, Canada, and European countries [4,5].

The nitrosamine contaminants that prompted the US FDA recall announcements are NDMA, N-nitrosodiethylamine (NDEA), N-nitroso-N-methyl-4-aminobutyric acid (NMBA; N-nitrosomethyl-3-carboxypropylamine), and N-nitroso-varenicline. The first two have long been recognized as carcinogens by the International Agency for Research on Cancer (IARC), the United States Environmental Protection Agency (US EPA), the National Toxicology Program (NTP), and California’s Proposition 65 [6,7,8,9,10] (Table 1). The third contaminant, NMBA, induces bladder and kidney tumors in rats [11,12]. While there no carcinogenicity data are available for N-nitroso-varenicline, N-nitroso compounds as a group have been identified as a “cohort of concern” in internationally harmonized guidance on impurities in pharmaceuticals, based on their mutagenic properties and carcinogenic potencies [13].

Multiple causes can lead to nitrosamine contamination of drugs [14,15,16,17,18]. Nitrosamines can form from precursors under certain conditions during the manufacturing process, e.g., the simultaneous presence of a secondary, tertiary or/and quaternary amine and nitrite under acidic reaction conditions. Additional sources of contamination can stem from the use of recovered reagents (e.g., solvents, catalysts) that have been comingled from different processes or across manufacturing lines without control and monitoring, and from the use of raw materials that have become contaminated due to inadequate cleaning of equipment.

The detection and elimination of nitrosamine contaminants poses a challenge as “typical tests for API (active pharmaceutical ingredient) purity, identity, and known impurities are unlikely to detect the presence of a nitrosamine impurity” [19]. In response to this situation, US FDA published guidance for industry on how to reduce potential nitrosamine formation from various sources and manufacturing processes [15]. The level of nitrosamine contaminants present in the finished product can also increase over time under normal storage conditions, and can increase more significantly when stored at temperatures higher than room temperature (as has been reported for the drug ranitidine) [20]. These conditions have resulted in unacceptable levels of NDMA in the drug ranitidine and have led to the market withdrawal of all prescription and OTC ranitidine-containing products in the US [20]. While some aspects of this problem, such as source of contamination and mechanisms of formation as well as some risk estimates have been discussed elsewhere [16,17,18,21], we calculated cancer risk estimates using chemical-specific cancer potencies developed by different agencies [8,9,22,23]. We further illustrate the significant carcinogenic potential of additional N-nitrosomethyl-*n*-alkylamines (NMAs) besides NDMA; these NMAs are closely related to several other contaminants identified to-date and if present in medications, would raise additional concerns.

## 2. Public Health Concerns

Carcinogenic nitrosamine contaminants have been detected in medications that include first-line treatments for prevalent chronic conditions such as hypertension (ARBs), heartburn (histamine-2 blockers), and type 2 diabetes (metformin). This is a public health concern, given that tens of millions of Americans with these conditions may have been treated with affected medications. For example, hypertension is estimated to affect 116 million American adults, with the highest prevalence in the Black population [24], and over 83.4 million prescriptions for ARBs were issued in 2018 [25]. The second group of affected medications, histamine-2 blockers, had over 25.2 million prescriptions issued in the US in 2018, in addition to its availability in a number of OTC preparations [26]. Histamine-2 blockers are a first-line treatment option for medical management of heartburn, which is estimated to affect at least 60 million Americans once a month and 15 million Americans on a daily basis [27]. The use of the antihyperglycemic drug metformin is similarly widespread, with nearly 83.8 million prescriptions written for metformin hydrochloride alone in the US in 2018 [28]. Metformin is a first-line treatment for type 2 diabetes, which is estimated to affect as many as 34.2 million Americans, with higher prevalence in nonwhite populations [29]. Metformin is also increasingly prescribed for treatment of endocrine, cardiovascular, and other metabolic disorders [30].

Given the extensive use of these medications in the US, there is widespread potential for exposure to unacceptable levels of carcinogenic nitrosamine contaminants if the contamination is not controlled. Additional consideration is warranted for individuals taking more than one such medication simultaneously for management of multiple conditions, which may result in increased exposure to these carcinogenic contaminants across different medications. Additionally, individuals may be more or less susceptible to development of cancer as disease results from a combination of fixed and variable intrinsic factors (e.g., sex, epigenome, nutritional status) and potentially modifiable extrinsic factors (e.g., occupational exposures, racism and other psychosocial stressors, physical activity, diet) that comprise all biologically relevant external stressors [31]. The fact that some of these affected medications are first-line treatments for conditions that disproportionately impact populations with health disparities who may be more susceptible, should be of public health concern.

US FDA notes that nitrosamine impurities should not be present in drugs as they may increase the risk of cancer if people are exposed to them above acceptable levels and over long periods of time [19,32]. In 2019, the agency set interim acceptable daily intake limits for nitrosamines based on a cancer risk of 1 in 100,000, e.g., 0.096 μg/day for NDMA and 0.0265 μg/day for NDEA [33]. These levels attempt to “balance the risks of potential long term carcinogenic risk and disruption to clinical management of patients’ hypertension and heart failure” [19] and other conditions.

As evidenced by the large number of drug recalls, these interim levels have been exceeded numerous times, and discovery of additional nitrosamine-contaminated drugs has continued seemingly unabated. In this paper, we first provide a review of the evidence of carcinogenicity for these contaminants and other related nitrosamines. Next, to illustrate the potential public health impact, we use valsartan as a case study to estimate the additional cancer risk to individuals taking this medication, based on US data (i.e., US FDA-reported levels of NDMA and NDEA contamination) and likely exposure durations. A similar approach using European data has been taken by the European Medical Association [14].

## 3. Results

### 3.1. Evidence on the Carcinogenicity of NDMA, NDEA, NMBA, and the Larger Group of NMAs

Two nitrosamine contaminants, NDMA and NDEA, reported in certain drug products by US FDA since July 2018, are dialkyl nitrosamines. NDMA is also an NMA. As implied by the name, NMAs have a methyl and an alkyl group (methyl or greater) attached to the second nitrogen. Thirteen NMAs have been classified as carcinogens under Proposition 65 (Table 1). NDMA has two methyl groups and hence is the simplest member of the NMAs, while the structurally similar nitrosamine NDEA has two ethyl groups attached to the second nitrogen. A third nitrosamine contaminant identified in certain drug products, NMBA, is a common metabolite of NMAs with an alkyl group of four carbons (NMA-C4) or longer. Thus, NMAs represent a group of carcinogenic nitrosamines that are structurally and/or metabolically related to NDEA and NMBA.

The majority of carcinogenicity data for NDMA, NDEA, NMBA, and the larger group of NMAs stems from studies in laboratory animals [6,10,11,12,34,35]. In addition, long-term dietary epidemiological studies suggest that both NDMA and NDEA may increase pancreatic cancer, and NMDA may increase several types of gastrointestinal cancer in humans. However, a review of human epidemiological studies is beyond the scope of this article [10,36,37,38,39]. Below we review evidence on the carcinogenicity of NDMA, NDEA, NMBA, and the larger group of NMAs, focusing on findings from animal bioassays, metabolism studies, and genotoxicity assays.

#### 3.1.1. Animal Bioassays

Here we summarize data from animal studies of NMAs (including NDMA), NDEA, and NMBA conducted in a variety of laboratory animal species and strains. Studies of these nitrosamines often employed varied study protocols in terms of number of dose groups, number of animals per dose group, dose levels tested, exposure duration, and route of administration. NDMA and NDEA were the most studied of these nitrosamines, with bioassays using wide dose ranges and large numbers of animals per dose group [10,22,23,34]. For all chemicals, systemic tumors were induced via each route of administration tested (see details in Table S1 for characterization of the tumors).

As summarized in Table 2, each of these nitrosamines induced tumors at multiple organ sites in each of the species tested. Several of the tumor sites were common to different nitrosamines. In rats and hamsters, the nasal cavity, lung, and liver are the most frequently occurring tumor sites seen in studies of these nitrosamines. In mice, the lung and liver are the major target sites. In addition, each of these nitrosamines induced rare tumors in multiple organs of each of the species tested. In rats, these rare tumors occurred in the nasal cavity, tongue, oropharynx, lung, esophagus, forestomach, liver (cholangiocarcinoma and hemangiosarcoma), kidney, and urinary bladder. In hamsters, rare tumors occurred in the nasal cavity, lung, forestomach, liver, and urinary bladder, and in mice, rare tumors occurred in the nasal cavity, tongue, esophagus, and forestomach.

Most notably, for each of these nitrosamines short-term exposures (e.g., a single dose; once weekly doses for 25 weeks or less; daily doses for 8 weeks or less) resulted in the induction of tumors in dosed animals [6,34]. For example, NDMA induced lung and kidney tumors in mice exposed for just one week; similarly, a single administration of NDEA induced liver and kidney tumors in rats [34]. Overall, the results from animal studies show that these nitrosamines are potent carcinogens, with positive tumor findings in multiple organ/tissue sites across multiple species, including the induction of rare tumors [6].

#### 3.1.2. Metabolism

The metabolism of NMAs (including NDMA) and NDEA has been characterized through in vivo and in vitro studies [6,34,40,41].

Metabolism of nitrosamines involves denitrosation and hydroxylation, with common metabolites formed across rodent species [6]. Similar to many other nitrosamines, NDMA, NDEA, and the larger group of NMAs require metabolic activation for genotoxic and carcinogenic activity [42]. This activation is thought to occur primarily via cytochrome P450 (CYP)-dependent mixed function oxidase-catalyzed hydroxylation. Multiple cytochrome P450 enzymes are involved in the metabolism of NMAs, catalyzing demethylation, hydroxylation, dealkylation, and denitrosation reactions [6,43].

Hydroxylation at the alpha carbon results in formation of carbonyl compounds (including the carcinogens formaldehyde, in the case of NMAs, and acetaldehyde, in the case of NDEA) and an electrophilic intermediate, the alkyl-diazonium ion (see Supplementary Materials, Figure S1 and references [42,44]). The alkyl-diazonium ion has been proposed to react with DNA or other nucleophilic molecules, giving rise to alkylation products and one molecule of N_2_ [40,41,42]. Hydroxylation of NMAs at a nonalpha carbon ultimately yields a series of carboxylated and hydroxylated products, including the carcinogen N-nitrososarcosine (for NMA-C2 and NMAs with longer alkyl chains) [45], and the animal tumorigens N-methyl-nitroso-2-oxopropylamine (MOP) (for NMA-C3 and NMAs with longer alkyl chains) [11], 4-hydroxy-nitrosomethyl-*n*-butylamine [46], and NMBA (for NMA-C4 and NMAs with longer alkyl chains) (see Supplementary Materials, Figure S2, modified based on Huang et al., 1993) [47].

#### 3.1.3. Genotoxicity

The genotoxicity of the NMAs (including NDMA) and NDEA has been demonstrated in multiple assay systems, ranging from bacterial reverse mutation assays to in vivo and in vitro mammalian assays, while the testing of NMBA has been limited to genotoxicity assays conducted in *Salmonella* and yeast (Table 3). With the exception of NMA-C14, each of these nitrosamines has been tested in bacterial mutagenicity assays, and all induced mutations [6,34,48]. With the exception of NMBA and NMA-C14, each nitrosamine has been tested and shown to form DNA adducts in vivo [6,23]. NDMA, NDEA, and some of the other nitrosamines have been tested and shown to induce other markers of DNA damage, including DNA strand breaks and unscheduled DNA synthesis [6,23,49,50,51]. Only NDMA and NDEA were tested for chromosomal effects, with positive findings including chromosomal aberrations, sister chromatid exchange, and micronuclei formation [6,50,52]. A more detailed summary of the genotoxicity findings for these chemicals is provided in Table S2 [6,22,23,34,48,49,50,51,52,53,54,55,56,57].

### 3.2. Cancer Risk Estimate

To quantify the potential impact from taking these nitrosamine-contaminated medications, we calculated the cancer risk based on the nitrosamine levels reported by US FDA. While detections of NDMA and NDEA have been reported in a large number of drugs, contaminant levels have only been reported for a few, with levels varying among drug products (Table 4). In the case of NMBA, it has been detected in losartan and valsartan products, but concentrations of NMBA present in these products have not been reported. As shown in Table 4, the highest level of NDMA reported by US FDA was 20.19 μg per tablet in a valsartan product, and the highest level of NDEA was 1.31 μg per tablet, also in a valsartan product. These nitrosamine levels far exceed US FDA’s interim acceptable intake limits of 0.096 μg/day for NDMA and 0.0265 μg/day for NDEA [15,33].

In this case study the cancer risks from exposures to NDMA and NDEA are separately estimated for individuals taking valsartan, based on the following assumptions. For NDMA, the highest level was detected in 320 mg tablets of valsartan, and we assumed that individuals took one 320 mg valsartan tablet per day, with each tablet contaminated with 20.19 μg NDMA, for 6 years. For NDEA, the highest levels were detected in 160 mg tablets of valsartan, and we assumed that individuals took two 160 mg valsartan tablets per day, with each tablet contaminated with 1.31 μg NDEA, for 6 years. The valsartan dosage assumptions are consistent with the recommended maximum valsartan dose of 320 mg per day for treating hypertension and related heart conditions [61]. The use of 6 years as the duration of exposure to nitrosamine-contaminated valsartan assumes that exposure began in July 2012, when manufacturing processes changed [62], and continued until the first US FDA recall in July 2018.

In the risk estimates presented here, it is assumed that individuals are exposed to a single nitrosamine contaminant (i.e., NDMA or NDEA) from a particular drug product; however, multiple nitrosamines can be present in the same drug product. This case study also assumes that an individual takes only one type of nitrosamine-contaminated medication, although some individuals may take multiple types (e.g., ARBs and histamine-2 blockers).

The extra cancer risk accrued over a specified period of exposure to a nitrosamine-contaminated drug can be calculated as Equation (1) [63]:

(1) $$\mathrm{Extra}\;\mathrm{Cancer}\;\mathrm{Risk}=\mathrm{Cancer}\;\mathrm{Potency}\;\times \;\mathrm{Exposure}\;\mathrm{Rate}\;\times \;\left(\frac{\mathrm{years}\;\mathrm{exposed}}{70\;\mathrm{years}}\right)$$

where 70 years is the assumed human lifetime.

The exposure rate is calculated using Equation (2):

(2) $$\mathrm{Exposure}\;\mathrm{Rate}=\left[\frac{\mu g\;\mathrm{carcinogen}}{\mathrm{tablet}}\;\times \;\frac{\mathrm{tablets}}{\mathrm{day}}\;\times \;\frac{0.001\;\mathrm{mg}}{\mu g}\right]\;/\;70\;\mathrm{kg}$$

where 70 kg is the assumed human body weight.

#### 3.2.1. Extra Cancer Risk Calculation for NDMA

The cancer potency for NDMA is estimated as 16 (mg/kg/day)^−1^ by California’s Proposition 65 program [22] and 51 (mg/kg/day)^−1^ by the US EPA [8], both of which are based on liver tumor data in female rats [64].

Using the valsartan dosage assumptions presented above for NDMA, the exposure rate is:

(3) $$\left[\frac{20.19\;\mu g\;\mathrm{NDMA}}{\mathrm{tablet}}\text{×}\frac{1\;\mathrm{tablet}}{\mathrm{day}}\times \frac{0.001\;\mathrm{mg}}{\mu g}\right]/70\;\mathrm{kg}=0.000288\;\mathrm{mg}/\mathrm{kg}/\mathrm{day}$$

Using the cancer potency estimate of 16 (mg/kg/day)^−1^, the extra cancer risk accrued as a result of exposure for 6 years to one daily 320 mg tablet of valsartan, where each tablet contains 20.19 μg NDMA is:

(4) $$16\;{\left(\mathrm{mg}/\mathrm{kg}/\mathrm{day}\right)}^{-1}\;\times \;0.000288\;\mathrm{mg}/\mathrm{kg}/\mathrm{day}\;\times \;\left(\frac{6\;\mathrm{years}}{70\;\mathrm{years}}\right)=4\;\times \;10^{-4},\;\mathrm{or}\;40\;\mathrm{per}\;100,000$$

A similar calculation using the cancer potency estimate of 51 (mg/kg/day)^−1^ for NDMA shows that the extra cancer risk is:

(5) $$51\;{\left(\mathrm{mg}/\mathrm{kg}/\mathrm{day}\right)}^{-1}\;\times \;0.000288\;\mathrm{mg}/\mathrm{kg}/\mathrm{day}\;\times \;\left(\frac{6\;\mathrm{years}}{70\;\mathrm{years}}\right)=1.26\;\times \;10^{-3},\;\mathrm{or}\;126\;\mathrm{per}\;100,000$$

#### 3.2.2. Extra Cancer Risk Calculation for NDEA

The cancer potency for NDEA is estimated as 36 (mg/kg/day)^−1^ by California’s Proposition 65 program [23] and 150 (mg/kg/day)^−1^ by the US EPA [9], based on liver tumor data in female rats [64].

Using the valsartan dosage assumptions presented above for NDEA, the exposure rate is:

(6) $$\left[\frac{1.31\;\mu g\;\mathrm{NDEA}}{\mathrm{tablet}}\;\times \;\frac{2\;\mathrm{tablets}}{\mathrm{day}}\;\times \;\frac{0.001\;\mathrm{mg}}{\mu g}\right]\;/\;70\;\mathrm{kg}=0.0000374\;\mathrm{mg}/\mathrm{kg}/\mathrm{day}$$

Using a cancer potency of 36 (mg/kg/day)^−1^, the extra cancer risk accrued as a result of exposure for 6 years to two daily 160 mg tablets of valsartan, where each tablet contains 1.31 μg NDEA, is:

(7) $$36\;{\left(\mathrm{mg}/\mathrm{kg}/\mathrm{day}\right)}^{-1}\;\times \;0.0000374\;\mathrm{mg}/\mathrm{kg}/\mathrm{day}\;\times \;\left(\frac{6\;\mathrm{years}}{70\;\mathrm{years}}\right)=1.2\;\times \;10^{-4},\;\mathrm{or}\;12\;\mathrm{per}\;100,000$$

A similar calculation using a cancer potency of 150 (mg/kg/day)^−1^ shows that the extra cancer risk is:

(8) $$150\;{\left(\mathrm{mg}/\mathrm{kg}/\mathrm{day}\right)}^{-1}\;\times \;0.0000374\;\mathrm{mg}/\mathrm{kg}/\mathrm{day}\;\times \;\left(\frac{6\;\mathrm{years}}{70\;\mathrm{years}}\right)=4.8\;\times \;10^{-4},\;\mathrm{or}\;48\;\mathrm{per}\;100,000$$

## 4. Discussion

NDMA, NDEA, NMBA, and the larger group of NMAs are potent genotoxic carcinogens, and nitrosamines should not be present at significant levels in medications. Alerted by the numerous recalls in several commonly used medications contaminated with nitrosamines, we conducted a case study to quantify the potential increased cancer risk to individuals taking these medications on a chronic basis. Using the maximum contaminant levels reported for NDMA and NDEA in certain recalled valsartan drug products, and assuming continuous exposure to these levels for six years, we estimated the range of cancer risks associated with these two nitrosamines using cancer potencies developed by California’s Proposition 65 program and US EPA. For NDMA, the estimated cancer risks ranged from 40 to 126 additional cancer cases per 100,000 exposed individuals. For NDEA, the estimated cancer risks ranged from 12 to 48 additional cancer cases per 100,000 exposed individuals. Using another approach, US FDA [58] has also estimated the additional lifetime risk for patients taking the maximum daily dose of valsartan for four years to be one extra cancer in 8000 individuals for NDMA (equivalent to 12.5 in 100,000) and one extra cancer in 18,000 individuals (equivalent to 5.6 in 100,000) for NDEA. US FDA’s assumptions in estimating risks include a 4-year exposure duration and a human body weight of 50 kg. All of these cancer risk estimates exceed US FDA’s generally accepted lifetime cancer risk of 1 in 100,000 for impurities in pharmaceutical products [13].

While the exposure scenario selected for use in this case study is unlikely to reflect actual exposures, it is unclear if the selected scenario is more likely to result in an over- or an underestimate of the cancer risk from nitrosamine-contaminated medications. For instance, nitrosamine-contamination levels are likely to vary within the same drug product from batch to batch, and individual medication use patterns also change over time (e.g., duration, dose, addition of multiple nitrosamine-contaminated medications). Our case study calculated risks for NDMA and NDEA separately, assuming exposure to only one nitrosamine in a given drug product. We also assumed a stable level of nitrosamine contaminants in the finished drug product based on the maximum measured levels reported. In the absence of available data, we were unable to account for potential increases of nitrosamines that can occur with longer storage time and higher temperatures during both storage and distribution, as has been reported for NDMA. Nitrosamine contamination can occur in a broader range of medications and continues to be an issue, as indicated by the identification of N-nitroso-varenicline in the antismoking drug varenicline three years after the first recall of NDMA-contaminated ARB drugs [3,65]. This supports our concern that nitrosamine exposures may be even higher for specific populations taking multiple medications than assumed in our case study.

## 5. Conclusions

Our risk analyses underscore the importance of preventing nitrosamine contamination from occurring in widely used drugs, and the necessity of removing contaminated drug products from the market. Prompt manufacturing changes and continued monitoring by US FDA are needed to address this serious public health issue. Ultimately, the presence of carcinogenic contaminants in drugs may affect treatment preferences, patient compliance, and health outcomes.

## Figures and Tables

**Table 1:** Cancer classifications of some nitrosamines found in pharmaceutical drugs and structurally related N-nitrosomethylalkylamines (NMAs).

Nitrosamine	Structure	Cancer Classification ^1^
N-Nitrosodimethylamine (NDMA, NMA-C1)	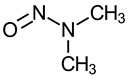	IARC 2A (1987) US EPA B2 (1986) NTP RoC RA (1981) P65 (1987)
N-Nitrosodiethylamine (NDEA)	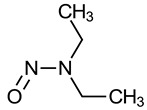	IARC 2A (1987) US EPA B2 (1986) NTP RoC RA (1981) P65 (1987)
N-Nitroso-N-methyl-4-aminobutyric acid (NMBA)	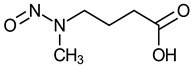	Not evaluated
N-Nitrosomethylethylamine (NMA-C2) N-Nitrosomethyl-*n*-propylamine (NMA-C3) N-Nitrosomethyl-*n*-butylamine (NMA-C4) N-Nitrosomethyl-*n*-pentylamine (NMA-C5) N-Nitrosomethyl-*n*-hexylamine (NMA-C6) N-Nitrosomethyl-*n*-heptylamine (NMA-C7) N-Nitrosomethyl-*n*-octylamine (NMA-C8) N-Nitrosomethyl-*n*-nonylamine (NMA-C9) N-Nitrosomethyl-*n*-decylamine (NMA-C10) N-Nitrosomethyl-*n*-undecylamine (NMA-C11) N-Nitrosomethyl-*n*-dodecylamine (NMA-C12) N-Nitrosomethyl-*n*-tetradecylamine (NMA-C14)	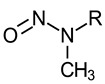 R: alkyl group (where C2 indicates a 2 carbon alkyl group, C3 indicates a 3 carbon alkyl group, and so on)	NMA-C2: IARC 2B (1987) US EPA B2 (1988) P65 (1989) NMA-C3 through NMA-C12, and NMA-C14: P65 (2014)

^1^ Cancer classification by various authoritative bodies. International Agency for Research on Cancer (IARC) Group 2A: probably carcinogenic to humans; IARC Group 2B: possibly carcinogenic to humans; US Environmental Protection Agency (US EPA) Group B2: probable human carcinogen; National Toxicology Program (NTP) Report on Carcinogens (RoC) RA, reasonably anticipated to be a human carcinogen; P65, California Proposition 65 (The Safe Drinking Water and Toxic Enforcement Act of 1986) carcinogen.

**Table 2:** Summary of statistically significant tumors and increased rare tumors in rats, hamsters, and mice exposed to NMAs (including NDMA), NDEA, and NMBA.

Tumor Site	Nasal Cavity			Tongue			Oropharynx			Lung			Esophagus			Forestomach			Liver			Kidney			Bladder			Others		
Species	R (r)	H (r)	M (r)	R (r)	H	M (r)	R (r)	H	M	R (r)	H (r)	M	R (r)	H (i)	M (r)	R (r)	H (r)	M (r)	R (r) ^1^	H (r)	M	R (r)	H	M	R (r)	H (r)	M	R	H	M
NDMA ^2^	X	X								X		X					X		X	X	X	X	X	X				X^3^	X^3^	X^3^
NDEA ^2^	X	X	X		X			X^4^		X	X	X	X	X	X		X	X	X	X	X	X						X^5^	X^5^	X^5^
NMBA ^6^																						X			X					
NMA-C2 ^7^	X	X*								X			X						X	X*								X^8^		
NMA-C3 ^9^	X*	X*	X*	X			X				X*	X*	X*			X			X*	X*	X*							X^10^	X*^,10^	X*^,10^
NMA-C4 ^9^	X	X*		X			X				X*		X*			X	X*			X*										
NMA-C5 ^9^	X*	X*		X*		X					X*	X*	X*	X	X*	X	X*	X^11^		X								X*^,12^		
NMA-C6 ^9^	X	X		X						X*^,13^	X*		X*^,13^			X	X*		X*^,12^	X*						X				
NMA-C7 ^9^	X	X*		X						X*^,13^	X*		X*^,13^				X*		X*^,12^	X*										
NMA-C8 ^9^	X*	X								X*	X*						X*		X*	X*					X*	X		X*^,14^		
NMA-C9 ^9^	X									X*									X*											
NMA-C10 ^9^	X									X*						X									X*					
NMA-C11 ^9^										X			X			X			X											
NMA-C12 ^9^		X								X*	X*		X			X*									X*	X*		X*^,15^		
NMA-C14 ^9^										X												X			X*					

R (rat); H (hamster); M (mouse); “X” denotes observation of tumors, “blank” denotes no tumor was observed; “gray” denotes not tested; * statistically significant (*p* < 0.05) increases of tumor incidence by Fisher pairwise comparison; (r) = rare tumor; (i) = infrequent tumor. ^1^ Cholangiocarcinoma and hemangiosarcoma of the liver are rare in rats; ^2^ Tumors reported by NTP (2016) [10], IARC (1978) [34], statistical significance information of tumor incidence was not provided; ^3^ Bile duct (rat) (NTP 2016, IARC 1978) [10,34]; blood vessel (hemangioma or hemangiosarcoma) (rat, hamster, mouse); ovary (female hamster) (NTP, 2016) [10]; ^4^ Larynx, pharynx (NTP 2016, IARC 1978) [10,34]; ^5^ Upper respiratory tract, thymus (thymoma), upper digestive tract, and mammary gland (benign adenoma) (rat); cheek pouch, trachea, and bronchi (hamster); respiratory tract, upper digestive tract, and leukemia (mouse) (IARC 1978; NTP 2016) [10,34]; ^6^ Lijinsky et al. (1983) [11]; Thomas et al. (1988) [12]; ^7^ US EPA (2003) [35], OEHHA (2014) [6]; ^8^ Leukemia (rat) (US EPA 2003) [35]; ^9^ OEHHA (2014) [6]; ^10^ Epiglottis (r) (rat); larynx-trachea-bronchial tract*, digestive system* and thyroid* (hamster); larynx-trachea-bronchial tract* (mouse); ^11^ Esophagus and forestomach combined; ^12^ Trachea* (r) (rat); ^13^ No concurrent control, but tumor incidence ≥ 90%; ^14^ Trachea* (r), and liver hemangiosarcomas (r) (rat); ^15^ Pancreas (rat).

**Table 3:** Summary ^1^ of genotoxicity findings for NMAs (including NDMA), NDEA, and NMBA.

Chemical	Mutagenicity ^2^	DNA Damage and/or Nucleic Acid or Protein Binding ^3^	Chromosomal Effects ^3^
NDMA	+Rodent mutation assays (in vivo and in vitro); +SLRL mutation assay in *Drosophila*; +*Salmonella* and *E. coli*; +*S. cerevisiae*	+DNA breaks in human cells (in vitro); +DNA breaks in rodent tissues (in vivo); +UDS in human and rodent cells (in vitro); +DNA adducts in several human cells and tissues (in vitro) and in rodent tissues (in vivo)	+MN in rodent cells and tissues (in vivo and in vitro); +CAs in rodent cells (in vitro); +SCE in rodent tissues (in vivo)
NDEA	+Rodent mutation assays (in vivo and in vitro); +SLRL mutation assay in *Drosophila*; +*Salmonella* and *E. coli*; +*S. cerevisiae* and *Neurospora crassa*	+DNA breaks in human cells (in vitro); +UDS in rat cells (in vitro); +DNA adducts in several human cells and tissues (in vitro); +DNA, RNA adducts in rodent tissues (in vivo)	+CAs and +SCE in CHL cells (in vitro)
NMBA	+Yeast; +*Salmonella*	NT	NT
NMA-C2	+CHL mutation assay; +Salmonella	+DNA breaks in human cells (in vitro); +DNA adducts in rodent tissues (in vivo)	NT
NMA-C3	+CHL mutation assay; +*Salmonella* and *E. coli*	+DNA adducts in rat tissues (in vivo)	NT
NMA-C4	+*Salmonella* and *E. coli*	+DNA, RNA, protein adducts in rat tissues (in vivo)	NT
NMA-C5	+*Salmonella*	+DNA adducts in rat cells and tissues (in vivo and in vitro); +8-oxodG in rat tissues (in vivo)	NT
NMA-C6–NMA-C12	+*Salmonella*	+DNA adducts in rat tissues (in vivo)	NT
NMA-C14	NT	NT	NT

^1^ See Table S2 for references and additional information on the genotoxicity findings from studies of the NMAs (including NDMA), NDEA, and NMBA. ^2^ Some negative or equivocal findings in *Salmonella* assays have been reported for NDMA, NDEA, and NMBA; however, the overall evidence indicates that NDMA, NDEA, and NMBA are mutagenic in one or more strains of *Salmonella*. ^3^ Some negative findings in assays of DNA damage and chromosomal effects have been reported for NDMA and NDEA; however, the overall evidence indicates that NDMA and NDEA cause these effects. “+” denotes positive results, NT denotes not tested; SLRL mutations: sex-linked recessive lethal mutations; SCE: sister chromatid exchange; CAs: chromosome aberrations; MN: micronucleus; UDS: unscheduled DNA synthesis; CHL: Chinese hamster lung V79 cells; 8-oxodG: 8-oxo-7,8-dihydro-2′-deoxyguanosine.

**Table 4:** Reported levels of nitrosamines in certain drug products (US FDA ^1^).

Drug Class and Major Indication	Active Pharmaceutical Ingredient (Dose per tablet)	NDMA (μg/tablet)	NDEA (μg/tablet)
Angiotensin II receptor blockers (ARBs): hypertension and related heart conditions	Valsartan (160 mg) *	0.45	1.31
	Valsartan (320 mg) *	<LOD–20.19	<LOD–1.22
Histamine-2 blockers: heartburn and gastroesophageal reflux disease (GERD)	Ranitidine (75 mg) **	0.01–0.04	NR
	Ranitidine (150 mg) **	0.01–0.33	NR
	Ranitidine (300 mg) **	0.01–0.86	NR
	Nizatidine (150 mg)	0.01–0.02	NR
	Nizatidine (300 mg)	0.01–0.03	NR
Antihyperglycemic: type 2 diabetes	Metformin, extended release (500 mg) *	<LOD–0.19	NR
	Metformin, extended release (750 mg) *	0.01–0.08	NR
	Metformin, extended release (1000 mg) *	<LOD–0.01	NR
	Metformin, immediate release (500 mg)	<LOD	NR
	Metformin, immediate release (850 mg)	<LOD–0.01	NR
	Metformin, immediate release (1000 mg)	<LOD	NR

LOD: limit of detection; NR: not reported; * recalled; ** withdrawn from the market. ^1^ Information presented in the table is from US FDA as of July 2021 (See References [58,59,60]).

## Data Availability

Not applicable.

## Supplementary Materials

The following are available online at https://www.mdpi.com/article/10.3390/ijerph18189465/s1, Figure S1: Metabolic activation of alkyl-nitrosamines and formation of an alkyl-diazonium ion, Figure S2: Proposed routes of NMA-C4 metabolism (Modified based on Huang et al., 1993 [47]), Table S1: Characterization of tumors (local vs. systemic) in rats, hamsters, and mice exposed to NMAs (including NDMA), NDEA, and NMBA by various administration routes, Table S2: Additional information on the genotoxicity of NMAs (including NDMA), NDEA, and NMBA. References [6,7,23,34,47,48,49,50,51,52,53,54,55,56,57] are cited in the Supplementary Materials.
