# Supplementary material for: Estimated Cancer Risks Associated with Nitrosamine Contamination in Commonly Used Medications
Source: Int J Environ Res Public Health. 2021 Sep 8;18(18):9465. doi: 10.3390/ijerph18189465 (PMC8467924; doi:10.3390/ijerph18189465)
Supplement: Supplementary file 1 [file ijerph-18-09465-s001.zip › ijerph-1342988-supplementary.pdf]

Supplemental Materials

Estimated cancer risks associated with nitrosamine contamination in commonly used medications

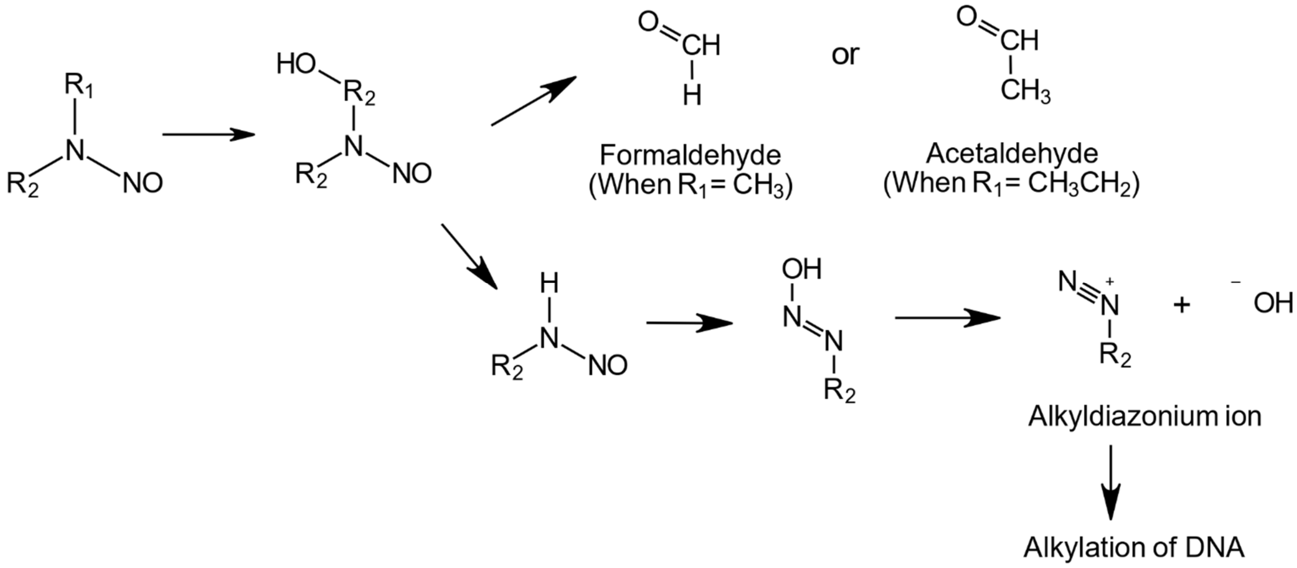

Figure S1. Metabolic activation of alkyl-nitrosamines and formation of an alkyl-diazonium ion.

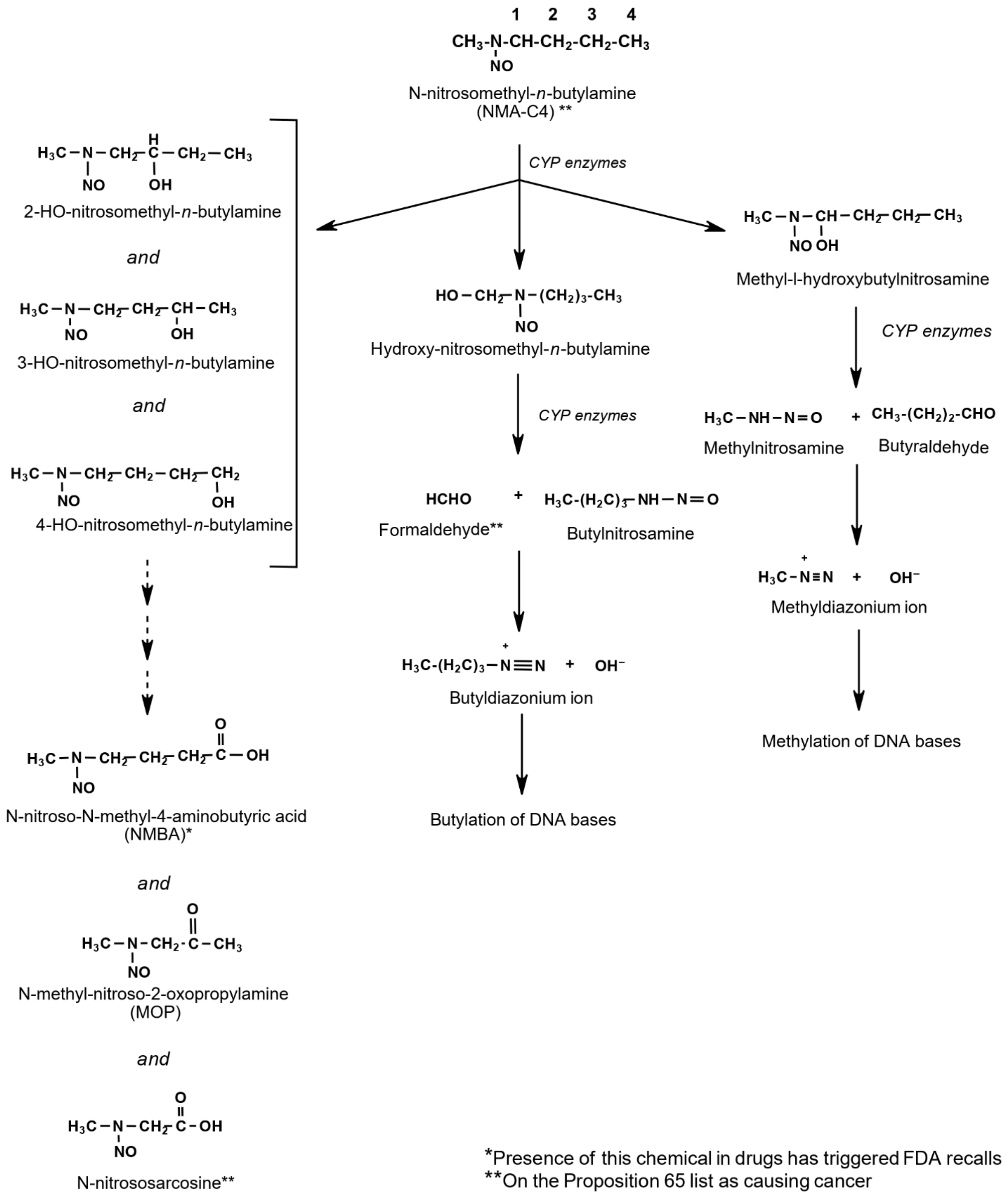

Figure S2. Proposed routes of NMA-C4 metabolism (Modified based on Huang et al., 1993 [47]).

NMA-C4 can be hydroxylated by CYP enzymes at the alpha carbon of either the methyl (center portion of figure) or alkyl group (right-hand portion of figure). This step leads to the formation of a hydroxy methyl alkyl nitrosamine followed by the formation of a mono alkyl nitrosamine and aldehydes. The mono alkyl nitrosamine spontaneously decomposes to form diazonium ions, which then can alkylate DNA. CYP enzymes can also oxidize NMAs at the non-alpha carbon of the alkyl chain (left-hand portion of figure). This leads to a variety of hydroxylated products and the downstream metabolites NMBA (N-nitrosomethyl-3-carboxypropylamine), MOP, and N-nitrososarcosine.

Table S1. Characterization of tumors (local vs systemic) in rats, hamsters, and mice exposed to NMAs (including NDMA), NDEA, and NMBA by various administration routes.

Routes	Oral			Inhalation			In Utero			Intraperitoneal Injection			Subcutaneous Injection			Others [1]		
**Species**	**R**	**H**	**M**	**R**	**H**	**M**	**R**	**H**	**M**	**R**	**H**	**M**	**R**	**H**	**M**	**R**	**H**	**M**
**NDMA**	S	L, S	S	L, S		L, S			S	S		S	S	S	S			
**NDEA**	L, S	L, S	L, S	S	L[2]		S	S	S	S	S	S		S	L, S	S (iv, ir)	S (sk)	S (sk)
**NMBA**	S															L, S (ives)		
**NMA-C2**	L, S	S																
**NMA-C3**	L, S	S						S					S	S	S			
**NMA-C4**	L, S	L, S														S (ives)		
**NMA-C5**	L[3]	L, S	L[4]							S	S	S	S			S (im)		
**NMA-C6**	L, S	L, S																
**NMA-C7**	L, S	L, S											S					
**NMA-C8**	S	L, S																
**NMA-C9**	S																	
**NMA-C10**	L, S																	
**NMA-C11**	L, S																	
**NMA-C12**	L, S	S												L, S				
**NMA-C14**	S																	

R (rat); H (hamster); M (mouse); gray denotes not tested; L: local tumors; S: systemic tumors; L, S: both local and systemic tumors.

[1] Other routes abbreviated in parenthesis are intravenous injection (iv), intravesicular injection in bladder (ives), intramuscular injection (im), intrarectal insertion (ir) and skin application (sk).

[2] Tracheal, bronchial, and lung tumors were observed in hamsters.

[3] Esophageal and forestomach tumors were observed in rats in drinking water studies.

[4] Lingual, esophageal and forestomach tumors were observed in mice in drinking water studies.

Table S2. Additional information on the genotoxicity of NMAs (including NDMA), NDEA, and NMBA.

Chemical Name (Acronym), CAS Number	Mutagenicity [1]	Chromosomal Effects [2]	DNA Damage/Nucleic Acid or Protein Binding [2]	References
N-Nitrosodimethylamine (NDMA or NMA-C1), 62-75-9	+*In vivo* transgenic rodent mutation assay; +Chinese hamster V79 and ovary cells; +Mouse lymphoma cells; +SLRL mutation assay in *Drosophila*; +*Salmonella*; +*E. coli*; +*S. cerevisiae*	+MN in rat hepatocytes, mouse bone marrow and hamster embryonic fibroblasts (*in vivo)*; +/−MN in rat bone marrow (*in vivo)*; +CAs in hamster embryonic fibroblasts (*in vivo*) and lung V79 cells (*in vitro*); +SCE in human lymphocytes and fibroblasts (*in vitro*), in mouse bone marrow (*in vivo*), in mouse hepatocytes, and Chinese hamster ovary cells, V79 cells and primary lung cells (*in vitro*); +/−SCE in Chinese hamster bone marrow (*in vivo*)	+DNA strand breaks in mouse and rat liver, kidney (*in vivo)*; +DNA strand breaks in human lymphoma cells (*in vitro)*; +DNA adducts in several human cells (*in vitro*); +DNA adducts in rat liver, lung, kidney (*in vivo)*; +UDS in human fibroblasts and fetal mouse kidney and liver, mouse testes, rat liver, respiratory cells (*in vitro*)	OEHHA 2006 [50]; OEHHA 2014 [6]; OEHHA 1988 [22]; Morita et al. 2016 [52], Kawaguchi et al. 2018 [49]
N-Nitrosodiethylamine (NDEA), 55-18-5	+*In vivo* transgenic rodent mutation assay; +Chinese hamster V79 cells; +*Salmonella TA100, 1530*; +*E. coli*; -*In vivo* dominant lethal mutations in mice; +*In vivo* SLRL mutation assay in *Drosophila;* +*S. cerevisiae;* +*Neurospora Crassa*	+/−MN in mice (*in vivo*); +CAs in Chinese hamster cells (*in vitro*); +SCE in Chinese hamster cells (*in vitro*)	+DNA strand breaks in human lymphoma cells (*in vitro)*; +UDS in rat liver cells (*in vitro)*; +DNA adducts in human bronchus, colon and esophageal tissues (*in vitro*); +DNA, RNA adducts in rat, hamster and mouse liver, kidney (*in vivo)*	IARC 1978 [34]; OEHHA 1988 [23]; Morita et al. 2016 [52]; Kawaguchi et al. 2018 [49]; Yamazaki et al. 1985 [51]

Chemical Name (Acronym), CAS Number	Mutagenicity [1]	Chromosomal Effects [2]	DNA Damage/Nucleic Acid or Protein Binding [2]	References
N-Nitroso-N-methyl-4-aminobutyric acid (NMBA), 61445-55-4<br>(metabolite of NMA-C4 and NMAs with longer alkyl chains)	+Yeast *D7-144* and *RMO52*; +*Salmonella* TA1530; −*Salmonella* TA1535	NT	NT	Mehta and von Borstel 1984 [53]; Lijinsky and Andrews 1983 [54]; CCRIS 2018 [48]
N-Nitrosomethylethyl-amine (NMA-C2), 10595-95-6	+Chinese hamster V79 cells; +*Salmonella*	NT	+DNA strand breaks in human lymphoma cells (*in vitro)*; +DNA adducts in rat liver, lung, kidney (*in vivo)*	IRIS 1987 [55]; OEHHA 2014 [6]; Kawaguchi et al. 2018 [49]
N-nitrosomethyl-*n*-propylamine (NMA-C3), 924-46-9	+Chinese hamster V79 cells; +*Salmonella TA1530, 1535*; +*E. coli WP2 uvrA*	NT	+DNA adducts in rat esophagus, liver, lung, kidney (*in vivo)*	OEHHA 2014 [6]
N-nitrosomethyl-*n*-butylamine (NMA-C4), 7068-83-9	+*Salmonella TA100, 1535*; +*E. coli WP2 uvrA, hcr*⁻	NT	+DNA adducts in rat esophagus, liver, lung, kidney (*in vivo)*; RNA and protein adducts in rat liver, kidney, pancreas, spleen (*in vivo)*	OEHHA 2014 [6]
N-nitrosomethyl-*n*-pentylamine (NMA-C5), 13256-07-0	+*Salmonella TA1535*	NT	+DNA adducts in rat esophagus, nose, liver, trachea, lung, forestomach, kidney (*in vivo)*; DNA adducts in rat esophagus, liver (*in vitro)*; +8-oxodG in SD rats (*in vivo)*	OEHHA 2014 [6]; Bonde et al. 2007 [56]; Mirvish et al. 1995 [57]
N-nitrosomethyl-*n*-hexylamine (NMA-C6), 28538-70-7	+*Salmonella TA1535*	NT	+DNA adducts in rat liver (*in vivo)*	OEHHA 2014 [6]

Chemical Name (Acronym), CAS Number	Mutagenicity [1]	Chromosomal Effects [2]	DNA Damage/Nucleic Acid or Protein Binding [2]	References
N-nitrosomethyl-*n*-heptylamine (NMA-C7), 16338-99-1	+*Salmonella TA1535*	NT	+DNA adducts in rat liver (*in vivo)*	OEHHA 2014 [6]
N-nitrosomethyl-*n*-octylamine (NMA-C8), 34423-54-6	+*Salmonella TA1535*	NT	+DNA adducts in rat liver (*in vivo)*	OEHHA 2014 [6]
N-nitrosomethyl-*n*-nonylamine (NMA-C9), 75881-19-5	+*Salmonella TA1535*	NT	+DNA adducts in rat liver (*in vivo)*	OEHHA 2014 [6]
N-nitrosomethyl-*n*-decylamine (NMA-C10), 75881-22-0	+*Salmonella TA1535*	NT	+DNA adducts in rat lung, liver (*in vivo)*	OEHHA 2014 [6]
N-nitrosomethyl-*n*-undecylamine (NMA-C11), 68107-26-6	+*Salmonella TA1535*	NT	+DNA adducts in rat lung, liver (*in vivo)*	OEHHA 2014 [6]
N-nitrosomethyl-*n*-dodecylamine (NMA-C12), 55090-44-3	+*Salmonella TA100*	NT	+DNA adducts in rat lung, liver (*in vivo)*	OEHHA 2014 [6]
N-nitrosomethyl-*n*-tetradecylamine (NMA-C14), 75881-20-8	NT	NT	NT	OEHHA 2014 [6]

[1] Some negative or equivocal findings in *Salmonella* assays have been reported for NDMA, NDEA, and NMBA; however, the overall evidence indicates that NDMA, NDEA, and NMBA are mutagenic in one or more strains of *Salmonella*.

[2] Some negative or mixed findings in assays of DNA damage and chromosomal effects have been reported for NDMA and NDEA; however, the overall evidence indicates that NDMA and NDEA cause these effects.

"+" denotes positive results, "–"denotes negative results, "+/–" denotes some positive and some negative results, NT denotes not tested

SLRL mutations: Sex-linked recessive lethal mutations

SCE: Sister chromatid exchange

CAs: Chromosome aberrations

MN: Micronucleus

UDS: Unscheduled DNA synthesis

8-oxodG: 8-oxo-7,8-dihydro-2'-deoxyguanosine
